# Research Progress and Perspective on Drought Stress in Legumes: A Review

**DOI:** 10.3390/ijms20102541

**Published:** 2019-05-23

**Authors:** Muhammad Nadeem, Jiajia Li, Muhammad Yahya, Alam Sher, Chuanxi Ma, Xiaobo Wang, Lijuan Qiu

**Affiliations:** 1 School of Agronomy, Anhui Agricultural University, Hefei 230036, China; rananadeem.aaur@yahoo.com (M.N.); lijia6862@ahau.edu.cn (J.L.); aligenetics08@gmail.com (A.S.); machuanxi@ahau.edu.cn (C.M.); 2 School of Life Sciences, Anhui Agricultural University, Hefei 230036, China; genetics08248@gmail.com; 3 National Key Facility for Crop Gene Resources and Genetic Improvement, Institute of Crop Science, Chinese Academy of Agricultural Sciences, Beijing 100081, China

**Keywords:** legumes, drought stress, drought tolerance, QTLs, omics, CRISPR-Cas9

## Abstract

Climate change, food shortage, water scarcity, and population growth are some of the threatening challenges being faced in today’s world. Drought stress (DS) poses a constant challenge for agricultural crops and has been considered a severe constraint for global agricultural productivity; its intensity and severity are predicted to increase in the near future. Legumes demonstrate high sensitivity to DS, especially at vegetative and reproductive stages. They are mostly grown in the dry areas and are moderately drought tolerant, but severe DS leads to remarkable production losses. The most prominent effects of DS are reduced germination, stunted growth, serious damage to the photosynthetic apparatus, decrease in net photosynthesis, and a reduction in nutrient uptake. To curb the catastrophic effect of DS in legumes, it is imperative to understand its effects, mechanisms, and the agronomic and genetic basis of drought for sustainable management. This review highlights the impact of DS on legumes, mechanisms, and proposes appropriate management approaches to alleviate the severity of water stress. In our discussion, we outline the influence of water stress on physiological aspects (such as germination, photosynthesis, water and nutrient uptake), growth parameters and yield. Additionally, mechanisms, various management strategies, for instance, agronomic practices (planting time and geometry, nutrient management), plant growth-promoting *Rhizobacteria* and arbuscular mycorrhizal fungal inoculation, quantitative trait loci (QTLs), functional genomics and advanced strategies (CRISPR-Cas9) are also critically discussed. We propose that the integration of several approaches such as agronomic and biotechnological strategies as well as advanced genome editing tools is needed to develop drought-tolerant legume cultivars.

## 1. Introduction

Environmental stress factors, namely, heat, salinity, and drought, affect almost all aspects of the plant ranging from germination to the maturity stage [1,2,3,4]. Drought is a major threat and the most unpredictable constraint, with adverse effects on crop production worldwide [5,6,7]. Drought induces several devastating effects on plants by disturbing various plant activities such as the carbon assimilation rate, decreased turgor, increased oxidative damage, and changes in leaf gas exchange, thereby leading to a reduction in yield [7,8]. Plant sensitivity to drought is a complex phenomenon and depends on numerous factors including the growth stage of the plant, genetic potential, duration and severity of stress [9]. Drought also affects the leaf development, activity of enzymes, ion balance, and ultimately leads to yield reduction [6,10].

Legumes are a nourishing and low-cost source of protein, which play a vital role in agriculture due to their atmospheric nitrogen-fixation ability [11,12]. These distinct characteristics broaden their adoptability to environments that have nitrogen deficiency [13]. Legume crops are vulnerable to several abiotic threats, and drought has been known as a main constraint restraining crop yield [14,15,16]. Legume crops are commonly grown in rainfed regions, and different models (Global Climate Model) have predicted increases in the frequency and intensity of drought, indicating the threat of water scarcity [17]. Water deficiency at any stage can affect plant growth as a result of reducing crop production, especially during grain filling and the reproductive phase [18,19]. Drought frequency and severity limit grain yield, plant biomass and related components of legumes [20,21,22,23,24]. The extent of decrease in yield depends on the intensity and duration of drought stress (DS), crop developmental stage and genotypic variability. Therefore, the development of new approaches to improve drought tolerance in legumes is critical for reducing yield losses in water-deficient environments. Development of drought-tolerant varieties with improved water use efficiency (WUE) may lead to enhance crop productivity in dry areas [25].

The substantial development and integration of advanced approaches for dry environments are the primary elements that contribute to enhanced legume productivity in harsh environments. Approaches such as the development of various traits for drought tolerance, innovative breeding and water efficient practices, for instance, the use of drip irrigation and mulching, are promising ways to mitigate the devastating effects of drought [26]. The adverse effects of drought on several other crops have been previously reviewed [5,27,28,29], but no updated and comprehensive study is available on impacts of DS in legume crops. Our study about effects, mechanisms and management strategies may lead to managing the devastating effects of DS and to develop drought-tolerant genotypes in dry environments.

## 2. Effect of Drought Stress (DS) on Legumes

**Figure 1:**
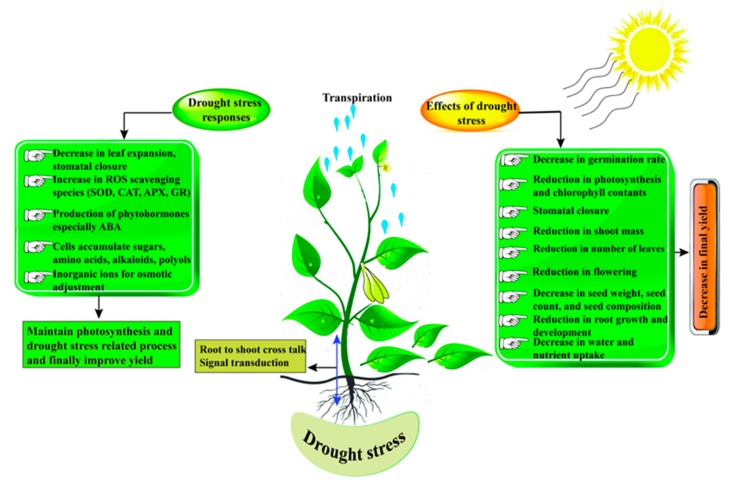
Effect of drought stress (DS) on plants and possible responses.

Legumes vary in their responses/sensitivity at the onset of drought, but in all cases, final yield is significantly decreased. This is linked with reduced germination and declined photosynthetic activity [8], decreased assimilate translocation and carbon fixation [30,31], repressed flowering time and an effect on reproductive organs [32], pollen grain sterility [33], fewer pods and lower grain set [34,35,36] and declined sink activity [37]. Drought affects several aspects of legume growth and development, including germination, shoot and root development, photosynthesis, and the reproductive stage. Due to global climate change, drought has become one of the most uncontrolled and an unpredicted factor which is continuously limiting crop production and has adverse effects on legume crops (Figure 1). Studies showed that severe drought conditions disturb plant morphology, physiology, and growing period, whereas moisture contents play an essential role in enzyme activation during germination which could help to elucidate the sensitivity of plants to drought at the germination stage. Germination and reproductive stages are highly sensitive to water deficit. Under DS, the germination rate was significantly reduced in soybean [38]. Awari and Mate [39] noted a decrease in the germination rate in chickpea under a water deficit. Li et al. [40] observed that DS commonly occurs at the seedling stage and significantly reduced yield in faba bean.

Photosynthesis is a fundamental process responsible for growth and development in plants and is influenced by various environmental stresses [41]. The intensity, rate, and duration of stress will affect plant responses to water deficit. DS affects carbon fixation by disturbing enzymatic activities of different enzymes such as PPDK, PEPCase, EBPase and Rubisco [42]. DS affects the photosynthetic machinery and ultimately decreases legume yield depending on the severity and extent of the stress. Ohashi et al. [43] observed that the photosynthetic rate, stomatal conductance, and transpiration rate significantly declined during DS. Similarly, a recent study reported a decline in net photosynthesis in soybean under drought, which caused a reduction in dry matter accumulation and the podding rate, directly decreasing the yield [44]. Hao et al. [45] reported that the chlorophyll content of drought-stressed soybean plants was reduced by 31% compared to non-stressed plants. In faba bean, DS considerably reduced the chlorophyll content, photosynthesis rate and impaired plant growth and yield [46]. Abid et al. [47] reported that drought influences chlorophyll fluorescence and antioxidant enzyme activities in faba bean. Likewise, in chickpea, DS affects the chlorophyll content, chlorophyll fluorescence and photosynthesis [48].

Moreover, stomatal control is also considered as a main physiological factor for optimizing water use during DS [49], preventing excessive water loss under extended drought conditions. For instance, stomatal conductance as compared to the control decreased by 60% under DS in soybean [45,50,51]. Abdel and Stutzel [52] reported that the stomatal conductance declined as the DS progressed, confirming the conclusion that the impact of drought was more significant under severe stress than under medium stress. Atti et al. [53] observed a decline in the photosynthetic rate, transpiration rate, and stomatal conductance by 78.4%, 85.4%, and 92%, respectively, during DS. They explained that the stomatal conductance was correlated with the transpiration rate more than with the photosynthetic rate, which was reported earlier [54]. The transpiration rate decreased by 53% [45] and 57% [50] under DS; this decrease was due to the decline of stomatal conductance which was controlled by root-originated ABA, as 50-fold xylem ABA was measured under drought conditions [55], with a significant increase as the stress became severe [35].

The impact of drought on final yield is very complex and comprises different processes such as fertilization, gametogenesis, embryogenesis and grain formation [56]. During the plant life cycle, flowering and reproductive phases are highly vulnerable to water scarcity [15,32]. DS affects flowering time and flower development and leads to pollen grain sterility by decreasing the growth of pollen tubes and pollen grain germination [15,57,58]. Drought severely affects the ability of the plant to produce more flowers, pods and seed set; thus, the final yield is ultimately decreased [19]. For instance, the pods per plant were reported to be reduced under water deficit by many scientists [22,50,59,60]. At the beginning of pod development, DS reduced pod number by 92.7%, while during pod lengthening, the reduction was 81.6% compared to controls, due to the cumulative effects of a reduction in pod induction, young pod abortion [61], pod enlargement [53], and to the reduction in flower number [62]. In previous studies, lower seed number per plant under DS was recorded [22,60,62]. The highest reduction in seed numbers per plant occurred in the flowering stage [63]. José et al. [64] reported that drought occurrence at flower formation led to a shorter flowering period and produced fewer flowers, fewer pods, and consequently, a smaller number of seeds per plant. However, it was concluded earlier that DS during the seed set period reduces seed number [65,66], and ultimately reduces final yield (Table 1). A more recent study observed a significant decrease in seed yield (73–82%) per plant in soybean under DS [67]. Farooq and co-workers reviewed the impacts of DS on legumes during the grain filling stage. They reported that drought is harmful in certain developmental stages, including the generation and function of reproductive organs and reported a 27–87% yield reduction [16]. For instance, water deficit significantly reduces yield in mash bean [68], soybean [69], and in chickpea [32].

## 3. Tolerance Mechanisms

To increase legume productivity under DS, it is imperative first to understand tolerance mechanisms. Plants have evolved several adaptations including escape and avoidance, compatible solute accumulation, antioxidant regulation, and hormonal regulation. Research progresses elucidating these mechanisms are discussed below.

### 3.1. Drought Escape and Avoidance

Drought escape (DE) is a primary adaptation mechanism which involves rapid plant growth and development to facilitate the completion of the life span prior to the onset of drought events. Legume crops can escape drought by shrinking their life span to avoid stress by retaining higher tissue water potential by improving water uptake and reducing water loss [85]. DE occurs when phenological development is successfully matched with periods of soil moisture availability, where the growing season is shorter and terminal DS predominates [56]. For instance, local cowpea cultivars flower prior along a transect from south to north through Sahelian and Sudanian regions of Africa headed to the Sahara desert [86]. Flowering time coincides with the time of cessation of the rainy season, which is an adaptive approach. Early flowering and seed set before an upcoming drought event is an important trait in legumes and cereals [27,87]. Legume crops with an indeterminate growth habit (such as common bean and cowpea) may mitigate the adverse effect of short-term DS by producing new organs during the phase of stress recovery [88]. The plants having a deep rooting system and a perennial growth habit have better capability to withstand stress than annuals with shallow-root systems [8]. However, if drought occurs at earlier stages, DE plants can slowly switch to drought avoidance with the succulent strategy or with a more progressive drought tolerance mechanism such as production of osmolytes and high WUE [89].

### 3.2. Solute Accumulation

Compatible solute accumulation is a fundamental strategy for osmoprotection and osmotic adjustment under DS. These compatible solutes accumulate primarily in drought-stressed cells without interfering with the macromolecules and are either hydroxyl compounds like oligosaccharides, polyhydric alcohols and sucrose or nitrogen-containing compounds such as amino acids and proline, polyamines and ammonium compounds [90]. The mechanism of osmoprotection is based on the close association of non-toxic elements with numerous components of the cell, whereas osmotic regulation assists in maintaining turgor through maintaining the water contents of cells [91]. During DS, proline plays an important role and act as a signalling compound to regulate mitochondria function and affect cell proliferation by means of activating particular genes, which are essential for stress recovery [92]. Proline accumulation aids in retaining membrane integrity by decreasing oxidation of lipids through guarding cellular redox potential and scavenging free radicals [93]. Among compatible solutes, non-reducing sugars, particularly di, tri and tetra-saccharides assist in maintaining the integrity of membranes [94]. For instance, Mannitol assists in stabilizing structures of macromolecules such as glutathione, ferredoxin, thioedoxin, phosphoribulokinase, and scavenging hydroxyl radicals [95,96]. Similarly, trehalose helps to stabilize macromolecules (e.g., membrane lipids, protein) and biological structures, thereby helping to improve photosynthetic activity under drought [97,98]. The defensive mechanism formation seems to be a consequence of hydrogen bonds among osmolytes and macromolecules, thus avoiding the creation of intramolecular hydrogen bonds that might irreversibly amend the three-dimensional structure of molecules. In legumes, the increased sugar alcohol (inositol and sorbitol) with a concomitant decline in sugar is the main osmoticum under water deficit [99].

### 3.3. Antioxidant Defense

**Figure 2:**
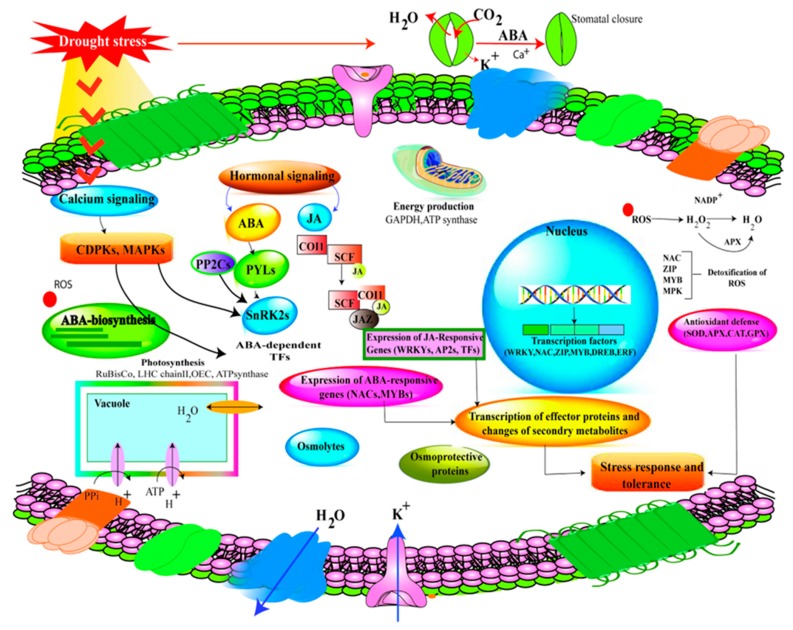
Schematic representation of drought tolerance mechanism in legumes. Reactive oxygen species (ROS), Ca^2+^, ABA, and JA are activated under DS. DS induces biosynthesis of ABA and JA, which, in turn, up-regulate the transcription of ion transporter genes. Overexpression of transcription factors (*WRKY*, *GmNACs*, DREB, *ZIP*, *AP2*/*ERF*, MYB) has been reported under DS. ABA, abscisic acid; JA, Jasmonic acid.

Reactive oxygen species (ROS) production is an initial response of drought-stressed plants and acts as a messenger to activate defense mechanisms in plant [100]. Under water deficit, ROS such as hydrogen-peroxide (H_2_O_2_), hydroxylradical (^•^OH), superoxide-radical (O_2_^•−^), and singlet-oxygen (^1^O_2_) and alkoxy radicals (RO) are produced and accumulate, which damage macromolecules and cell structure [101,102]. ROS acts as signalling compounds at low concentrations, and ROS trigger various responses under drought. When the level exceeds the defense mechanism, ROS cause oxidative stress to proteins, lipids and nucleic acids leading to altered intrinsic properties of biomolecules and cell death [103]. Enzymatic and non-enzymatic components regulate the defensive mechanism of ROS in the cells, and maintaining a higher concentration of antioxidants or antioxidant enzymes has proven to be an adoptive response under DS [104,105]. Enzymatic antioxidants comprise catalase (CAT), superoxide dismutase (SOD), glutathione peroxidase (GPX), ascorbate peroxide (APX), dehydroascorbate reductase (DHAR), glutathione reductase (GR) and monodehydroascorbate reductase (MDHAR) and non-enzymatic antioxidants include glutathione, ascorbate, tocopherols, carotenoids, phenolics and ascorbic acid [42,106]. Among enzymatic antioxidants, the SOD activity leads to detoxification of hydrogen peroxide (H_2_O_2_) and superoxide radicals [107]. APX helps to generate NADP^+^ and changes H_2_O_2_ to water [108] (Figure 2). APX also helps to remove H_2_O_2_ whereas DHAR and GR assist by providing a substrate for reactions. During oxidative stress, the concentrations of antioxidants may be increased more in the recovery phase than in the stress phase, as observed in green bean [109], pea [110,111], soybean [112], and chickpea [113]. Under DS, it been recorded that SOD, APX, GR, GST, GPX and POD activities increased in resistant cultivars of common bean and horse gram [114,115]. In conclusion, increased antioxidant activities in legumes would help to improve drought tolerance by protecting from oxidative stress.

### 3.4. Hormone Regulation

The phytohormones (including gibberellins, cytokinins, auxins, ABA and ethylene) regulate and control all aspects of plant growth and development. These plant hormones are involved in drought tolerance [116]. For instance, the rise in cytokinin level under water deficit in xylem sap stimulates stomatal opening by declining its sensitivity to ABA [117]. The concentration of gibberellins, cytokinins and auxin declines under water deficit while ethylene and ABA tend to increase in plants [118]. Under DS, the rise in ABA concentrations is due to a decrease in ABA catabolism hindering its entrance from the phloem and rhizosphere. Enhanced xylem pH in water deficit conditions also triggers ABA entry to root xylem [119]. For instance, declined stomatal conductance was linked with an increase in ABA concentration triggered by re-watering in kidney bean [120]. ABA also promotes hydraulic conductivity of roots which is accountable for increased water uptake and transport in plants [121,122]. ABA also increased the production of superoxide radicals and H_2_O_2_, enhancing the activities of antioxidant enzymes such as GR. Thus, ABA-induced gene overexpression can serve as a beacon of hope to improve drought tolerance. In addition, jasmonic acid (JA) is also essential to mitigate DS (Figure 2). However, JA usually cross-talks with other hormones to enhance survival of plants under water deficit. JA acid improves drought tolerance in plants by various means, including root development, scavenging of ROS, and stomatal closure [116]. In soybean, methyl jasmonate (MeJA) enhances drought tolerance and improves plant growth [123].

### 3.5. Potential Traits for Screening Legumes for Drought Resistance

The ever-increasing water shortage and frequent drought spells in agricultural ecosystems have been causing significant yield losses for many crops worldwide. Great efforts and substantial progress have been made through innovative research findings and rapid development of many novel techniques and methodologies in drought-resistance breeding. However, accumulated knowledge about drought-resistance in field crops as well in legumes is quite limited so far, and we still know little about the complex genetic architecture of drought tolerance and need to reveal the genetic bases of any trait associated with drought-resistance in crops, which can be applied in crop breeding [124]. Various traits have been used to screen for DS tolerance, including smaller leaf area, leaf area maintenance, water use efficiency, root and shoot biomass, osmotic adjustment, pod number per plant, and 100-grain weight (Table 2).

Among many factors that are strongly associated with drought tolerance in legumes, architecture of roots is one of the most promising traits for drought escape and could be used positively in drought tolerance breeding programs [125]. This aims to improve drought-resistance, enabling the plant to mine water efficiently from deeper soil layer under catastrophic dry environments and could be introduced or manipulated by a single gene [126]. For example, in soybean, experiments suggested that roots and root nodules are indispensable sensors of drought tolerance, and the feedback of these crucial organs on drought tolerance is the key feature. Direct screening of roots and nodule traits in the field along with identification of genes, proteins and metabolites will be necessary in order to gain a comprehensive and thoughtful understanding of regulation of root architecture [127]. In the context of drought-root cohesive bonds, this was investigated in common bean (*Phaseolus vulgaris* L.), and revealed negative impacts of drought on bean roots growth and ultimately decreased reproduction. This implies the existence of a core relationship between root traits and reproductive growth. Results showed reduction in rooting depth (14%), root biomass (29%), total root length (35%), volume (41%), pod set percentage (53%), and pod weight (43%) and illustrate how DS effects on root and shoot traits and pod set percentage in common bean, and root traits have a correlation with reproductive success under drought. Thus, DS adversely impacts bean yield, with severity of its intensity dependent on time duration, type, and plant growth stage as we have also discussed earlier. Therefore, root traits could be included in legume DS breeding programs [126].

In another study, 12 chickpea (*Cicer arietinum* L.) genotypes were evaluated under drought for root traits, and root: shoot ratio (RSR) was estimated. Huge variations were observed for RLD, RDW, deep RDW and RSR under drought conditions. Results depicted progressive contributions of RLD (after 45 DAS), deep RDW in maturity and RSR from early pod filling stages to yield under drought. Ramamoorthy and co-worker concluded that breeding for the more perfect combination of profuse RLD (at surface soil depths), and RDW (at deeper soil layers), would be best selection strategy, for efficient water use and boosted terminal drought tolerance in chickpea [128]. In mungbean (*Vigna radiate* L.), where twenty-five genotypes were tested under DS treatment at vegetative and reproductive stages. There is a significant decline in the relative water content (RWC), membrane stability index (MSI), proline content of leaves, leaf area plant height, and yield. They investigated direct links of these traits to drought tolerance as varieties which have retained high in values of RWC, MSI, protein, proline content, leaf area, plant height, and yield traits were identified as drought tolerant [129].

Moreover, slow canopy wilting (SW) has also a significant importance in drought tolerance. SW is a water conservation trait controlled by quantitative trait loci (QTLs) in plants, for instance, late maturity group of soybeans (*Glycine max* L. Merr.). Two exotic soybean landraces were identified as new SW lines in early maturity groups. They shared the same water conservation strategy of limited maximum transpiration rates. Yield trials of selected recombinant inbred lines from two top exotic crosses have also given the indication to support the advantage of SW in favor of drought resistance. Therefore, importance of SW under drought conditions provides a genetic means for improving drought tolerance in early maturity group soybean [130]. Generally, soybean cultivars vary in how swiftly they wilt in water scarcity situations and this pivotal trait may lead to improvement in yield under DS. Previously researchers designed an experiment to determine the genetic mechanism of canopy wilting in soybean and they used the plant material of a mapping population of recombinant inbred lines (RILs). They sum up that the genetic mechanism regulating canopy wilting is polygenic and environmentally sensitive and it would provide new insight in future research to scrutinize the genotypes for canopy wilting in drought tolerance of soybean and other legume crops [131].

Quantitative description of plant anatomical, ontogenetical, physiological and biochemical properties refers to plant phenotyping. Discovering and exploiting phenotyping traits also considerably contribute to drought tolerance in different legume crops. That has been witnessed in extra-early erect cowpea cultivars to escape terminal drought and should be recommended in regions with short rainfall periods [132]. Similarly, in climatic zones with limited rainfall in the middle of the growing season, delayed-leaf-senescence traits could be valuable characters for resistance to mid-season drought. Likewise, genetic mechanisms of early flowering and maturity time, seedling vigor, and high SPAD value (chlorophyll content), biological yield, and harvest index are exploited as primary traits for high seed yield in lentil in drought-prone environments [133]. In addition, early maturity (drought escape) and root trait (drought avoidance) drought tolerance in chickpeas and pigeon peas were also reported [134]. Furthermore, water use efficiency (WUE) is a key factor in determining crop yield, and is believed to relate to crop drought tolerance in many production systems. For example, in soybean, genotypes that possessed high WUE not only high yielded but also increased root penetrability of hardpans [135]. In conclusion, traits associated with drought tolerance in legumes could be a most promising way for positive use in drought tolerance breeding programs.

## 4. Management Strategies

To enhance legume productivity, concurrent development of drought-tolerant genotypes and practices for efficient water management are important. Therefore, to sustain and increase production of legumes under harsh environment, drought-tolerant genotypes and a site-specific package for production technology are required. Therefore, to meet the challenge of feeding a continuously growing population, scientists and breeders are looking for appropriate strategies to enhance legume productivity. The following sections will briefly review the various strategies for improving drought-tolerance of legumes under dry environments.

### 4.1. Agronomic Strategies

Agronomic approaches such as adjustment in time of sowing, plant geometry, and fertilizer management can help to improve drought tolerance [151,152]. The main influence of these management approaches to rainfall use efficiency is to improve total crop water use via transpiration and to lessen the loss of water by evapotranspiration and weeds. While chemical limitations to root growth are difficult to remediate, soil structure may be improved by the application of gypsum, which helps flocculate soil particles to enhance water infiltration and root growth [153]. Other agronomic practices such as mulching, zero tillage, and deep ploughing in the rainy season are critical agronomic practices to alleviate the adverse effects of DS in legumes [154,155]. In this regard, suitable cultivar selection is also critical.

#### 4.1.1. Planting Time and Plant Geometry

Managing the planting or sowing time can affect critical plant developmental stages such as flowering time and grain filling, thus mitigating the devastating effects of DS during these stages [156]. Early sowing with increased plant density can be a useful practice as it improves high rainfall use or water use efficiency to improve yield [157]. Legume crops can be successfully grown in dry areas by matching critical plant growth stages with the period of water availability to reduce final yield losses. Maintaining optimum plant density is vital for better use of natural resources such as water, light, space and nutrients. In rainfed areas, high planting density depletes moisture from soil prior to maturity, and more water will be lost by transpiration, whereas low planting density will leave unused soil moisture. Some scientists have observed that low plant density leads to low yield due to more pinched grains especially in Mediterranean environments [158]. Matsuo [159] studied the influence of plant density and row spacing on soybean growth and yield. They reported that plants produced at higher densities were taller, lodged more, were more sparsely branched, set fewer pods and seed than those plants at lower densities. Similarly, [160] observed that plant and row spacing improve WUE and yield components in soybean. Agajie et al. [161] studied the effect of spacing on yield components and yield of chickpea. They described that proper row spacing is critical for growth, yield components, and yield of chickpea. Thus, preferred plant density is a useful factor for high rainfall use efficiency and to obtain maximum yield per unit area.

#### 4.1.2. Nutrient Management

Managing micro- and macronutrient application is essential for developing drought-tolerant plants as better nutrition can efficiently mitigate the harmful effects of water deficiency [89,162]. Management of nutrient fertilizer has a positive influence on rain water use efficiency and hence on final yield. Both phosphorus and nitrogen fertilizers can improve crop water use efficiency and result in a decreased evaporation rate. For example, phosphorus nutrition improved photosynthesis, stomatal conductance, leaf water potential, membrane stability, and root development under drought in soybean [163]. Adequate K application increases photosynthesis efficiency in legume crops by maintaining higher tissue water potential during drought [164]. Nitrogen supply tends to enhance plant protein concentration, yield and yield-related traits in chickpea [165]. In soybean, application of inorganic fertilizers combined with farmyard manure enhanced organic carbon contents in soil; WUE and final yield increased by 76% and 103%, respectively, as compared to the control [166,167]. The productivity of legumes can be significantly enhanced by timely and proper irrigation particularly at critical stages, which can prevent pod abortion and decreased yield [168]. The addition of selenium (Se) improves water uptake ability of the root system during drought [56]. Mohammadi et al. [169] also reported that Se application reduces the lipid peroxidation and increases antioxidant enzymes activity such as GPX and SOD in chickpea and soybean under DS. Application of Se can promote the growth of ageing seedlings and delay leaf senescence [170]. Silicon (Si) application significantly improved the relative water content (RWC) in plants by improving the levels of glycine betaine and proline [171]. Moreover, Xu et al. [172] noted that Si addition can improve the tonoplast and plasma membrane structure in terms of integrity and function and chlorophyll florescence under water deficit. Si nutrition enhanced nodule activity for active fixation of nitrogen in cowpea [173]. In chickpea, addition of Si in combination with K significantly improved shoot dry matter during DS [174,175]. Zinc (Zn) application significantly increased yield attributes in drought-stressed chickpea [176]. Thalooth et al. [177] noted a significant improvement in growth, yield and related traits in mung bean with foliage application of zinc sulphate. Iron (Fe) and Zn addition can improve RWC and also have a positive impact on micronutrients and protein content of grains [178]. Boron is an important micronutrient, which is helpful in nitrogen fixation and nodule development. Boron foliar application promotes nodule formation in soybean during drought [179,180].

### 4.2. Plant Growth-Promoting Rhizobacteria and Arbuscular Mycorrhizal Fungal Inoculation

The use of plant growth-promoting *rizhobacteria* (PGPR) is a useful practice for alleviating the harmful effects of drought in legumes [181]. Application of PGPR enhances plant growth under drought through direct and indirect mechanisms [182,183,184], such as nitrogen fixation, phosphorus solublization, production of siderophores, organic acids and plant growth-promoting compounds as well as important enzymes such as ACC deaminase, glucanase and chitinase [182,185] (Table 3). PGPR can regulate the main phytohormones such as gibberellins, auxins, cytokinins, ABA and ethylene [186]. PGPR addition mitigates the harmful effects of drought to boost crop yield [187]. Dimkpa et al. [186] revealed that that inoculation with rhizobacteria (RBs) enhanced root hair development and lateral root, helping to improve water and nutrient uptake. 1-aminocyclopropane-1-carboxylic acid (ACC) hydrolysis by RBs hinders ethylene production and improves root growth in plants [188]. RBs such as *Bacillus*, *Burkholderia*, and *Arthrobacter* also promote proline synthesis under DS [189,190]. In soybean and cowpea, proton efflux activities in root systems were significantly and positively affected by *Azospirillum* inoculation [191]. Another study examined hormonal signalling mediated improvements in WUE, growth and yield in stressed pea upon application of ACC-deaminase activity containing *Variovorax paradoxus* [187]. Owing to ACC deaminase activity, RBs may convert ACC into a-ketobutyrate and ammonia, thereby shielding crop plants from harmful concentrations of ethylene [192].

Arbuscular mycorrhizal fungi (AMF) help to improve plant growth, yield, and uptake of water and nutrients under drought [193]. AMF can improve soil structure and soil water retention ability through stabilization and formation of soil aggregates. AMF produces a glycoprotein (Glomalin), which plays a vital role in improving soil structure [194]. The extra radical mycelium of AMF can explore and extend a large soil volume which assists in the better uptake of nutrients and water from the soil. Thus, AMF greatly assist in regulating tissue water potential, an avoidance mechanism to alleviate the detrimental impacts of water deficit on plant growth and development [195,196]. Additionally, inoculation with AMF can build up stress tolerance by increasing levels of osmoprotectants [195,196], decreasing lipid peroxidation, and increasing antioxidant potential [197,198], which ultimately boost final yield [196]. Gaur and Adholeya [199] observed improved plant growth and phosphate uptake in legume crops with AMF. A number of previous studies has witnessed that PGPR and AMF application have the ability to improve plant growth rate and crop yield under stress conditions by regulating hormonal and nutritional balances, solubilizing essential plant nutrients and producing plant growth regulators (Table 3). Besides the positive effects of sole inoculation of PGPR and AMF, their combined application also improves drought resistance. For example, Figueiredo et al. [200] observed the effect of application of *Paenibacillus polymyxa* and *Rhizobium tropici* on nodulation, N assimilation and growth in common bean under DS. Inoculation enhanced growth, nitrogen assimilation, and nodulation under water deficit compared with the control.

## 5. Development of DS-Tolerant Legumes Using Molecular and Biotechnological Approaches

To enhance legume productivity, concurrent development of drought-resistant genotypes and strategies for efficient water management is strategically important. Hence, integrated use of modern tools with conventional breeding protocols may produce significant benefits. This section discusses the breeding, molecular and transgenic approaches used to improve DS resistance in legumes.

### 5.1. Breeding Approach

Enhancing DS tolerance in plants through conventional breeding is a useful approach and a principal strategic for crop improvement [56]. However, selection and breeding approaches need substantial heritable variation to DS tolerance in legume crops [216,217]. Nevertheless, the breeding progress is often limited by quantitative genetic basis of traits and the inadequate knowledge of the physiological basis of crop yield response under drought [218]. In addition, heritability is often low due to high genotype and environment interactions, and variations in the amount and timing of precipitation received under dry conditions. Regardless of this, it is imperative to identify traits that confer yield potential and/or stability under DS. Furthermore, better characterization of the environment is a prerequisite to improve the effectiveness of target traits [219]. Screening and mass selection may be beneficial to achieve required phenotypic characteristics based on the traits correlated with the yield.

In legumes such as soybean, chickpea, common bean, and cowpea, certain root traits, for example, root length, fibrous root system, density and rooting depth are promising factors for DS avoidance [86,149,220], and may be useful for screening genotypes for DS tolerance. Traits such as early flowering, podding and maturity provide an escape mechanism, and may be used for mass screening [220]. Cooler canopies and high stomatal conductance have often been associated with higher grain yield under drought, and these traits possibly provide indirect selection criteria [220]. Canopy spectral reflectance is an effective non-invasive high-throughput phenotyping technique [221,222], enabling quick and easy measurements of several dynamic complex traits including carbon assimilation, biomass accumulation and plant canopy size [221]. Canopy spectral reflectance may, therefore, be used for mass screening of legume genotypes for drought resistance. Thermal infrared imaging (also called infrared thermography), which estimates leaf or canopy temperature, may also be employed to screen grain legumes for drought resistance [223]. Plant canopy temperature is a widely measured variable that is closely associated with canopy conductance at the vegetative stage and thus provides insight into plant water status [224]. Thermal infrared imaging for estimating conductance can be used at the whole plant or canopy level over time.

Evaluation of delayed senescence may be helpful for indirect selection of grain and biomass yields in breeding programmes targeting better drought tolerance [225]. Additionally, key physiological characteristics involving water use efficiency [226], root growth [227], carbon isotope discrimination (Δ^13^C) and leaf temperature [149] may be beneficial in screening legume genotypes for drought tolerance. Substantial genetic diversity in chickpea genotypes for carbon isotope discrimination [228] can be used for improvements of root architecture as an indirect indicator in chickpea. Wide hybridization is another strategy employed in breeding to achieve certain desirable traits within or between species. Interspecific crosses have been undertaken in many grain legumes with variable success [229]. In this perspective, *Phaseolus vulgaris* can be crossed with its wild relative *Phaseolus acutifolius*, which has a higher osmotic adjustment than the former, hence demanding its transfer to cultivated beans by interspecific hybridization [230]. Nonetheless, osmotic adjustment may have reduced stability depending upon the physiological stage of the plant or stress level [231]. Promising germplasm accessions have been developed in several legume crops in drought-related backgrounds, some of which have been found in chickpea (e.g., ICC 4958), related to root depth, root length density, terminal drought, and canopy temperature. Similarly, it has also been reported that wild genes were transferred into cultivated chickpea from *C. reticulatum*, resulting in nine genotypes well adapted to drought [232].

### 5.2. Quantitative Trait Loci (QTL) for Drought Tolerance

Genome-based approaches are valuable in finding desirable alleles, different QTLs having the potential to affect desired responses. Farooq et al. [56] reported that physiological and morphological traits, influencing the drought tolerance mechanism, are quantitatively inherited. Therefore, identification of QTLs related to drought tolerance is one of the most promising approaches using marker-assisted selection (MAS). Moreover, many breeding methodologies have been used in the improvement of drought tolerance in legumes based on MAS. Hamwieh et al. [233] identified 12 QTLs (NCPGR-50, TR-50, SCEA19, TAA-58, H6C-07, H5E-02, H5G-01, H6C-07, H1B-04, TA-113, H6C-07, H1F-21) related to seedling drought tolerance in chickpea. Radika et al. [234] reported the QTL *Qncl.Sw1* associated with grain yield in chickpea. In cowpea, Muchero et al. [235] reported seven markers ACC-3, VuPAT1-2, CPRD8- 1, CPRD14-2, CPRD14-3, CPRD22-2, CPRD22-4 linked with Dro-1, Dro-2, Dro-3, Dro-3, Dro-4, Dro-5, and Dro-5, respectively. Carpentieri-Pipolo et al. [236] identified four QTLs *qSV_Gm03*, *qSV_Gm05*, *qSV_Gm10*, *and qSV_Gm12* related to water deficit stress in soybean. QTL related to WUE and LASH (leaf ash) under terminal drought conditions in soybean were also identified [237]. Similarly, two QTLs were identified for both leaf ash and WUE, affecting root architecture, an important trait for adapting to drought [238]. Abdul-haleem et al. [239] reported five QTLs Gm01, Gm02, Gm03, Gm04 and Gm20 related to fibrous roots in soybean. Khazaei et al. [240] applied SNPs derived from *Medicago truncatula* L. to identify QTLs associated with stomatal characteristics in faba bean. Similarly, Mukeshimana et al. [241] used SNPs from BARCBean6K_3 Beadchip to identify 14 QTLs for traits related to drought tolerance in common bean. Furthermore, a recent study reported a cross between AND-277xSEA-5 used to map QTLs associated with stress tolerance to assess the factors that determine the magnitude of drought response in common bean [242]. They identified twenty-two QTLs for leaf and stem fresh biomass, chlorophyll, leaf temperature, leaf biomass dry weight, days to flowering, number of pods per plant, dry pod weight, number of seeds per plant, seed weight, and total yield under and drought and well-watered conditions.

### 5.3. Biotechnology and Functional Genomics

Through the advancement of crop transgenic tools, gene-based technology has appeared as a most valuable approach for comprehensive understanding of the complex mechanisms of resistance against drought and considered a complementary method for providing genetic modification in desirable plants. Recent progress in biotechnology enables us to identify specific genes that are resistant to abiotic stress from any other organism’s or even from different species to alter the genetic makeup of grain legume crops to protect against devastating drought conditions. Transgenic legumes can be transformed in a number of ways such as biolistic or agrobacterium-mediated transformation. In past studies, it has also been noticed that targeted resistant genes improved plant performance in drought environment without any negative impact on plant yield when it incorporated into various genomes. Many attempts are underway, but some experiments have already had success in different legume crops where transgenes have been designed by using diverse genes isolated within the genome as well as from other species.

Legume plants that were engineered based on single-gene transformation (Table 4), which encoding enzymes involved in the modification of membrane lipids and biosynthesis of osmoprotectants, and late embryogenesis proteins [243]. Many factors such as varying drought level, competency to transfer resistance mechanisms, and effect on plant yield and biomass are involved in controlling the whole process. Introduction of the osmoregulatory *P5CSF129A* gene into a chickpea genotype has been reported an increase in proline synthesis with a simultaneous decrease in malonaldehyde and free radicals levels, though there was no considerable rise in biomass accumulation [244]. Several genes belong to the AP2/ERF family, and DRED transcription factors have an integral role in plant growth and development, and they are considered pivotal in response to complex stress environments. Overexpressing *DREB1A* transgenic chickpea plants driven by the *Arabidopsis* rd29A promoter showed an increase in the expression of the *DREB1A* gene before 50% soil dehydration conditions [245]. Previous research revealed that the efficacy of rd29A: *DREB1A* on mechanisms underlying stomatal response, water uptake, rooting architecture, and transpiration efficiency under dry environments of plants, thereby imparting drought tolerance compared to controls. Li et al. [246] reported that overexpression *LOSS/ABA3* enhanced drought tolerance in soybean via enhancing ABA accumulation, which activates stress up-regulated gene expression and causes a series of biochemical and physiological resistance responses. Luchi et al. [247] observed that *VuNCED1* plays a key role in the synthesis of ABA in cowpea during drought.

### 5.4. OMICS-Based Approaches

**Figure 3:**
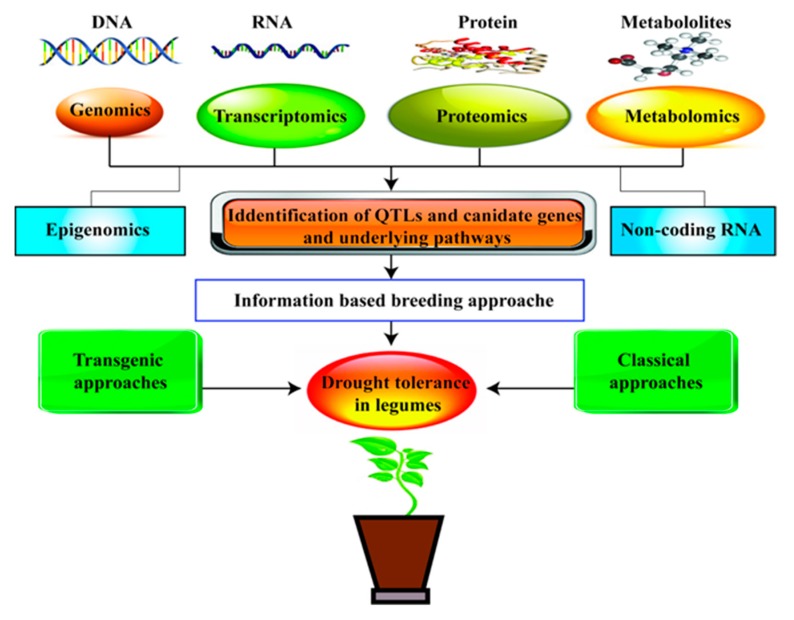
Schematic representation of the ‘omics’ approach for drought tolerance in legumes.

OMICS-based technology has been used to find out the desired trait genes and their specific function. This new approach uses transcriptome, genome, microme, proteome, and metabolome data (Figure 3) to locate candidate genes, thereby assisting in QTL mapping. Recently, scientific studies and research series are available to elucidate the functions of genes, proteins, and metabolites in legume sensitivity to DS. Another way to identify traits in OMICS approach is Phenomics; after launching Next-generation sequencing (NGS) a new era has started off transcriptomics-based sequencing of legumes. NGS approaches have been adapted to a wide variety of genome-scale surveys of sRNAs [279,280]. For instance, in soybean, a transcriptome atlas has been developed to perform RNA sequences of samples from 14 distinct drought-stressed conditions using the NGS approach [279]. Recently, Wang et al. [281] reported that RNA-sequencing assists in determining the transcriptional response of soybean to DS. In another study, comparative transcriptome analysis explicates the transcriptional alterations in drought-resilient and drought-sensitive soybean varieties under DS [282]. In chickpea, transcriptome analysis of oxylipin synthesis genes revealed early induction of jasmonate in roots under water deficit conditions [283]. Proteomics studies in soybean showed the presence of 35 proteins in roots under DS. Ferritin-type proteins that provide a defensive shield against oxidative stress expressed upregulation in roots under drought instead of the respective controls [284,285]. Likewise, drought-associated experiments were also conducted in chickpea, and SUPERSAGE analysis exposed root traits, and recognized 106 expressed sequence tags (EST)-based markers, unitags and SSR markers. ESTs serve as a source of high-quality transcripts for gene identification and development of functional markers associated with drought tolerance and may prove as a helping factor in breeding legumes for drought tolerance [286]. Pandey et al. [287] identified dehydration-responsive proteins in chickpea, which play a vital role in signal transduction and cell wall modification under DS. They reported 147 differentially expressed proteins and 205 differentially regulated protein spots found to have a function in nucleocytoplasmic transport, molecular chaperones, gene transcription and replication, chromatin remodeling, ROS pathway, and cell signaling. Similarly, [288] reported that some LEA proteins called dehydrins (CaN-600) were produced under stress, thereby protecting enzyme activity by scavenging ROS. Moreover, in many biochemical processes, proteomes are interlinked and will synthesize several metabolic products under drought. In a recent study in soybean, Das et al. [289] reported that metabolomic profiling revealed sugar and nitrogen metabolism have prime significance, along with phytochemical metabolism under water deficit conditions. In conclusion, the integration of such “omics” approaches would lead to drought-resilient legumes.

### 5.5. CRISPR/Cas9: Powerful Tool for Genome Editing (GE)

**Figure 4:**
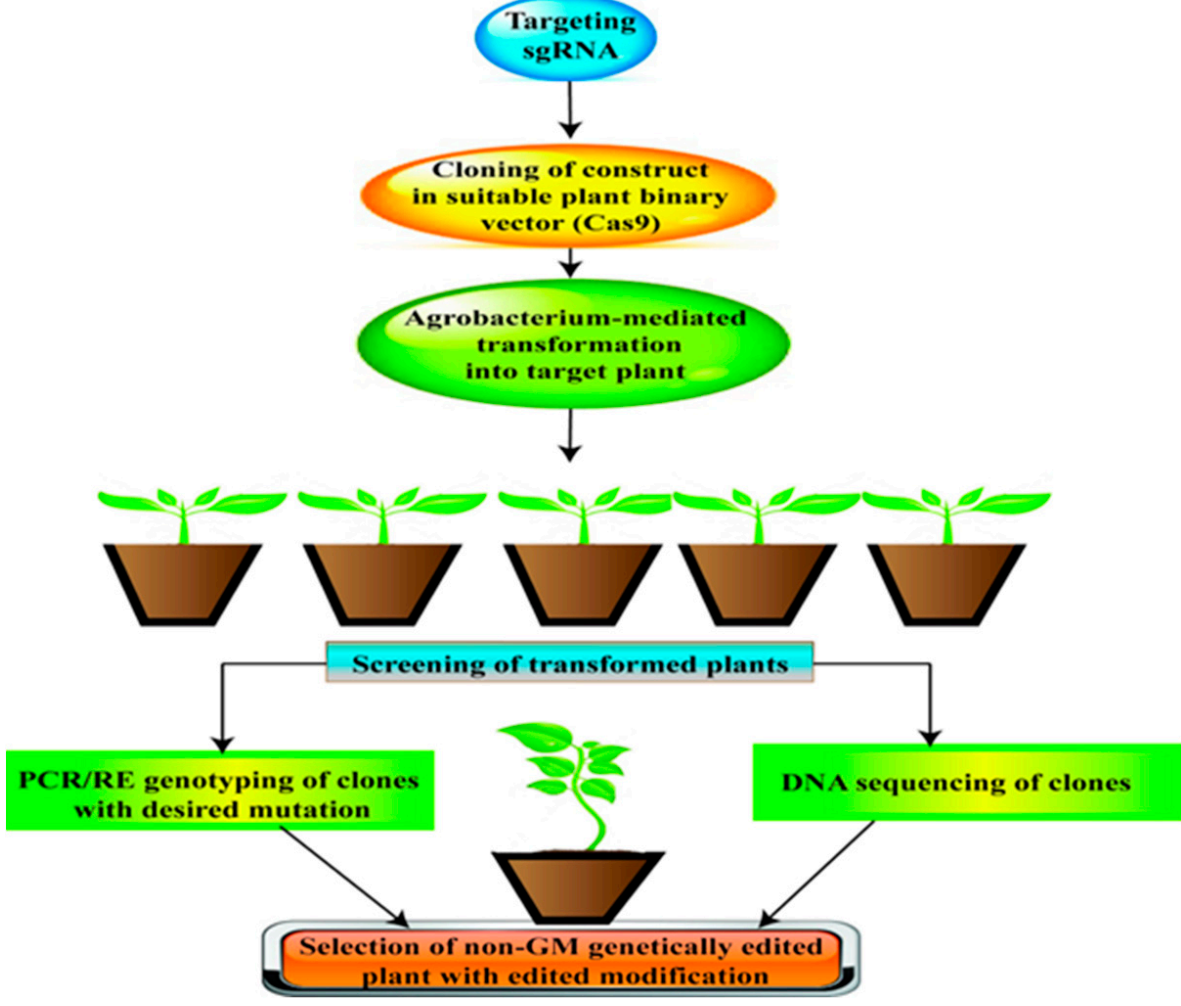
Schematic representation of an approach of genome editing (GE) with Cas9/sgRNA. First, the target gene is selected; sgRNAs are designed and synthesized using online tools. Generally, cloning of target sgRNA with Cas9 (or with its variant) is performed using a plant binary vector for *Agrobacterium*-mediated transformation into target plant species. Transformed plants are then selected for the presence of Cas9 and sgRNA, followed by PCR/RE genotyping. Finally, DNA sequencing is used for selecting the plants with the desired editing/mutation.

CRISPR/Cas9 is the most powerful and precise genome editing (GE) tool ever seen to date. Sustainable crop production under unpredictable environmental conditions is the most important objective of researchers, breeders and policymakers as they have to ensure food security in face of the rapidly growing human population. However, crop improvement through genetic recombination or random mutagenesis is quite laborious and cannot keep pace with rising food demands. CRISPR/Cas9 has opened up new possibilities to engineer any genomic sequence more efficiently with any target gene of interest. CRISPR/Cas9 leads to the development of non-genetically modified plants with desired traits that can contribute to enhance crop production under abiotic stress (Figure 4). In recent years, application of CRISPR/Cas9 has been reported in several crops: wheat [290], rice [291], barley [292,293], maize [294,295], and potato [296]. The recent reviews revealed the essential role of CRISPR/Cas9-mediated GE as a means to develop crops with improved tolerance to abiotic stresses [297,298]. Although only a few studies have adopted CRISPR/Cas9 for editing drought tolerance related genes have been reported in legumes, it has a vital role for future utilization in molecular breeding to enhance drought tolerance. Cai and co-workers first successfully achieved CRISPR/Cas9-mediated GE in soybean. They studied the efficiency of sgRNAs in hairy roots and used a single sgRNA for transgene (*bar*) and six sgRNAs that targeted various sites of two genes (*GmSHR* and *GmFE12*) in soybean [299]. In a most recent study, Cai et al. [300] reported CRISPR knockout of soybean gene (*GmFT2*) associated with flowering time; *GmFT2* mutants exhibiting late flowering under both short-day and long-day conditions. Hence, CRISPR/Cas9 GE for targeted and precise mutagenesis has huge potential in developing elite cultivars of legumes with durable and enhanced climatic resilience. In conclusion, CRISPR/Cas9 will be future of crop breeding as well as to target gene variation of complex quantitative traits, and thus will be the ultimate tool to maintain food security and provide relief from global hunger.

## 6. Conclusions and Future Research Perspectives

Given global climate change, sustainability of crop production is a serious challenging issue due to increasing incidences of both biotic and abiotic stresses in farmer’s field. Among the various abiotic stresses, DS is garnering serious attention, as it restricts plant growth and development and causes significant yield loss in legume crops, causing global food insecurity. DS negatively impacts overall plant growth ranging from the seedling stage to the reproductive stage and maturity stage. Key physiological, biochemical and metabolic pathways are seriously disrupted under DS, ultimately impacting plant performance negatively. In order to tackle the growing challenges of DS in legumes, several strategies could be employed. Exploration of untapped adaptive traits from various crop gene pools, and their precise incorporation into elite genotypes are urgently needed through pre-breeding activity and advanced breeding approaches. Likewise, physiological trait-based breeding approach as an alternative approach has tremendous potential for increasing the genetic gain under DS in legumes. Thus, these traits are receiving serious attention and are being incorporated in crossing programs to broaden the genetic base of legume varieties under various stresses including DS. Classical genetics and molecular-based breeding approaches, especially bi-parental family based QTL mapping, have shed light on the complex inheritance of drought tolerance in crop plants. In parallel, increasing efficiency of high-throughput genotyping platforms resulted in the release of draft genome sequence of various important global crops. Thus, this has allowed great opportunity to discover high-throughput markers for performing genome-wide association studies for investigating novel genomic variants related to drought tolerance existing across the crop genome. Moreover, emerging ‘omics’ sciences, including genomics, transcriptomics, proteomics and metabolomics could greatly improve our current understanding of the underlying drought-tolerant candidate genes and deciphering the intricate gene networks, and various signalling cascades involved in drought tolerance in legumes. Importantly, innovative techniques, viz., GE tools and ‘speed breeding’ will facilitate a deeper understanding and will effectively speed up the development of DS-resilient legumes to minimize the risk of global food insecurity.

## Figures and Tables

**Table 1:** Yield losses (%) in important legumes under drought stress (DS).

Legume Crops	Growth Stage	Yield Loss	Reference
Soybean	Pod set	73–82%	[67]
	Reproductive phase	46–71%	[70]
	Pod set	45–50%	[69]
	Grain filling stage	42%	[63]
Chickpea	Reproductive phase	45–69%	[71]
	Ripening stage	49–54%	[32]
	Anthesis stage	27–40%	[48]
	Ripening stage	50%	[72]
Cowpea	Reproductive phase	60%	[73]
	Reproductive phase	34–66%	[74]
	Pod filling stage	29%	[75]
Common bean	Reproductive phase	58–87%	[76]
	Pod filling stage	40%	[77]
	Flowering stage	49%	[78]
Pigeonpea	Reproductive phase	40–55%	[79]
Mung bean	Reproductive phase	26%	[80]
	Flowering stage	31–57%	[81]
Faba bean	Grain filling stage	68%	[82]
Lentil	Pod development	70%	[83]
	Reproductive phase	24%	[84]

**Table 2:** Potential traits/characters for screening legumes for drought resistance.

Legume Crops	Trait	Reference
Soybean	Water use efficiency, root architecture	[135]
	100-grain weight	[67]
	Lateral root thickness	[136]
	Presence of dense leaf pubescence	[137]
	Carbohydrate storage and remobilization	[138]
Chickpea	Prolific root system, Rooting depth, root length	[128,139]
	Shoot biomass, leaf area index, canopy temperature decrease	[140]
	Smaller leaf area	[141]
	Grain size, early maturity and short stature	[142]
Cowpea	Short duration and erect plant type	[86]
Common bean	Leaf RWC	[143]
	Deeper and vigorous roots	[144]
	Canopy biomass, pod partitioning index, stem biomass reduction and pod harvest index	[145]
Pigeonpea	Root and shoot biomass	[146]
	Leaf area maintenance	[147]
Mungbean	Dry matter partitioning	[148]
Faba bean	vigorous growth	[149]
	Root growth	[150]
Lentil	Dry root weight and root length	[133]

**Table 3:** Influence of arbuscular mycorrhizal fungi and rhizobacteria on drought resistance in grain legumes.

Legume Crops	Arbuscular Mycorrhizal Fungi/Bacterial Strains	Function	Reference
Soybean	*Bradyrhizobium japonicum*	Improve growth and yield	[201]
	*Bradyrhizobium japonicum*	Improved grain yield	[202]
	*Glomus mosseae, Glomus etunicatum*	Maintenance of high leaf water potential	[203]
	*Bradyrhizobium japonicum*	Improved N contents	[203]
	*Glomus intraradiecs*	Protected against oxidative stress and root osmotic adjustment	[195]
	*Pseudomonas cepacia*	Early growth and ACC-diaminase production	[204]
	*Bacillus spp.*	Enhanced nodulation and pod formation	[205]
Cow pea	*Azospirillum spp.*	Improve proton efflux activities	[191]
	*Glomus intraradiecs*	Improve Stomatal conductance	[206]
Common bean	*Paenibacillus polymyxa and rhizobium tropici*	Improved nodulation, N contents and plant growth	[200]
	*Glomus intraradiecs*	Maintain root hydraulic conductance	[207]
	Gigaspora margarita	Dehydration maintenance	[208]
	*Glomus intraradiecs*	Improve Stomatal conductance	[208]
	*Azospirillium brasilense*	Improve root growth	[209]
	*Bacillus spp.*	Improved nodulation and root hair proliferation	[210,211]
Green gram	*Glomus mosseae, Glomus intraradiecs*	Improved water-use efficiency	[212]
	*Pseudomonas putida*	Improved root growth and ACC-diaminase production	[213]
Pea	*Varovorax paradoxus*	Plant growth improvement through hormonal signaling	[187]
	*Pseudomonas spp.*	Alleviating drought stress	[214,215]
Lentil	*Pseudomonas putida*	Enhanced nodulation and plant growth	[214]

**Table 4:** Candidate genes explored for imparting drought resistance in legumes.

Legume Crops	Gene Transferred	Function	Reference
Soybean	*PgTIP1*	Confers drought tolerance	[248]
	*GmDREB2*	Enhance drought tolerance	[249]
	*GmRACK1*	Improve drought tolerance during vegetative growth	[250]
	*AtABF3*	Improve drought tolerance	[251]
	*GmFDL19*	Enhance drought tolerance	[252]
	*GmSK1*	Enhance tolerance to drought	[253]
	*GmNAC, GmDREB, GmZIP, ERF089*	Transcription factors	[238]
	*DREB1A, rd29A*	Transcription factors	[254]
	*GmBIN2*	Enhance tolerance to drought	[255]
	*GmCaM4*	Upreglate several drought-responsive genes	[256]
	*CDPK*	Enhance water permeability across the membrane	[257]
	*GmHK, GmCLV1A, GmCLV1B, GmRLK1, GmRLK2, GmRLK3, GmRLK4*	Osmosensor	[258]
Chickpea	*Aquaporins*	drought stress tolerance	[259]
	*DREB2A*	Transcription factors	[260]
	*MYB, WRKY, bZIP*	Transcription factors	[261]
	*MyB, AP2/ERF, XPB1*	Transcription factors	[262]
Cowpea	*VuPLD1, VuNCED1, CPRD8, CPRD12, CPRD14, CPRD22*	ABA-biosynthesis	[263]
Mungbean	*VrbZIP*	Drought-responsive gene	[264]
	*codA*	Improve abiotic stress tolerance	[265]
	*VrWRKY*	Enhance abiotic stress tolerance	[266]
Common bean	*Asr1, Asr2*	ABA signaling pathway	[267]
	*PvLEA3*	Protein stabilization	[268]
	*DREB2B*	Non-ABA dependent response	[267]
Pigeonpea	*C.cajan_29830, C.cajan_33874*	Improve drought tolerance	[269]
	WRKY, MyB, NF-Y	Transcription factors	[270]
Broad bean	*VfPIP1*	Aquaporin/water transport	[271]
Alfalfa	*AtEDT1*	Confers drought tolerance	[272]
	*SPL13*	Improve drought tolerance	[273]
	*CsLEA*	Enhance tolerance to drought	[274]
	*GsZFP1*	Confers drought tolerance	[275]
	*codA*	Enhance tolerance to drought	[276]
	*HaHB11*	Confers tolerance to water deficit	[277]
	*AVP1*	Enhance drought tolerance	[278]

## Abbreviations

GA	Gibberellin
ABA	Abscisic acid
PPDK	Pyruvate, phosphate dikinase
JA	Jasmonic acid
GB	Glycine betaine
ROS	Reactive oxygen species
GR	Glutathione reductase
GPX	Glutathione peroxidases
APX	Ascorbate peroxidase
SOD	Superoxide dismutase
GST	Glutathione S-transferases
MDHAR	Monodehydroascorbate reductase
CAT	Catalase
sRWC	Relative water content
WUE	Water use efficiency
PGPR	Plant growth promoting rhizobacteria
AMF	Arbuscular mycorrhizal fungi
WGS	Whole genome sequencing
SNP	Single-nucleotide polymorphism
SSRs	Simple sequence repeats
NGS	Next-generation sequencing
MDA	Malondialdehyde
GE	Genome editing
